# *Alzheimer's & Dementia—Translational Research and Clinical Interventions*: Strategic vision and expanded guidelines for manuscript submissions

**DOI:** 10.1002/trc2.12277

**Published:** 2022-03-15

**Authors:** Barry D. Greenberg

**Affiliations:** ^1^ Department of Neurology Johns Hopkins University School of Medicine Baltimore Maryland USA

As a specialty journal focused on the components of its descriptive title, *Alzheimer's & Dementia—Translational Research and Clinical Interventions* (*TRCI*) is in a unique position to strengthen the breadth of understanding and integration across the multidimensional topics that contribute to translational research and its outcomes in Alzheimer's disease and related dementias (AD&RD). Translational research is the nexus through which basic science and human studies merge. Its ultimate objective is the delivery of meaningful clinical outcomes to patients, based on an understanding of disease biology. A fulsome description of “translational research,” as has been published elsewhere (*e.g*., see Rubio et al.^1^) is beyond the scope of this editorial. Rather, the current objective is to summarize aspects of “translational research” in AD&RD that will be used to prioritize and solicit articles for *TRCI*, as a guideline for authors. The vision is based on the following three questions:

Basic science: What key biological mechanisms can be validated in hypothesis‐driven basic and preclinical research that make them suitable for advancement to clinical studies in humans?

Clinical studies: How can clinical studies be improved or made more informative to account for the inherent heterogeneity in dementia‐causing neurodegenerative diseases, ultimately delivering successful interventions that treat disease subtypes and further inform basic and clinical knowledge of disease mechanisms?

Diversity, inclusion, and health equity: How can diversity, inclusion, and health equity be incorporated from basic research to observational and interventional studies in humans, to broaden understanding of disease biology and its inherently confounding heterogeneous onset, progression, and clinical outcomes?

While these questions are seemingly independent, it is critical to understand how they inter‐relate. For example, basic science and clinical studies are interconnected by a two‐way vector, each informing the other in an iterative ongoing relationship. Moreover, the importance of relationships among the biology; clinical science; and social constructs of diversity, inclusion, and health equity has grown increasingly evident over recent years. Thus, these questions need to be considered embroidered into a “quilt” of translational research in ADRD. The expanded discussion below should enable submitting authors to consider how their research, their discussions, and their perspectives fit within the context of the journal's priorities.

## 1 BASIC SCIENCE

A basic science submission must fully recognize its limitations to be acceptable for publication in *TRCI*. For example, partial disease models in experimental animals can be useful for biomarker development and exploratory drug discovery issues such as target engagement, PKPD (pharmacokinetics–pharmacodynamics), ADMET (absorption, distribution, metabolism, excretion, toxicity), and response to therapeutic agents. However, translational value to human biology has been shown to be limited, particularly behavioral and cognitive measures, systems pharmacology, and other variables that confound their interpretation (*e.g*., strain, age, experimental controls, etc.). These issues will need to be fully addressed, elaborating on how they impact on interpretations of the study and its speculations to human disease.

To move beyond exclusive reliance on animal models, development and use of emerging human‐based model systems such as induced pluripotent stem cells (iPSCs) and three‐dimensional cell‐based model systems (*e.g*., microfluidic co‐cultures and organoids) will be encouraged. Knowledge gained through well‐designed studies will be useful in clarifying disease‐associated cellular mechanisms, as well as species‐specific differences in key biological systems such as inflammation, cholesterol, energy metabolism, and neuroanatomy. Recent single cell transcriptomic studies relating humans with rodent models certainly highlight these issues, further development of which will also be of interest to the journal.

Basic science submissions should also elaborate on how their outcomes will lead to clinical development studies. If this can't be convincingly done, there are better alternative journals for those sorts of submissions.

## 2 CLINICAL STUDIES

In coordination with *Alzheimer's & Dementia* and its sister open access journal *A&D‐DADM* (*Diagnosis, Assessment, and Disease Monitoring*), *TRCI* will welcome biomarker studies that inter‐relate with clinical/cognitive measures as part of deep endophenotyping that is critical to understanding the complexities of AD&RD biology, both in observational and interventional contexts. Well‐conducted studies of novel biomarkers or biomarker combinations that elucidate cellular, physiologic, or pathologic mechanisms that predict or contribute to disease onset and progression will be attractive to the journal.

In terms of clinical trials, customary enrollment criteria appear to be exquisitely defined, but in reality randomize heterogeneous cohorts to various treatment arms. It is anticipated that this heterogeneity is normalized by the randomization process, but this approach carries assumptions that are limited to “what we know” *a priori*. For example, responders and non‐responders have been observed within treatment arms in pivotal trials, but the information value of such observations can be lost if interpretations are based solely on comparisons of within‐arm statistical measures, perhaps contributing to ongoing failures in delivering on prespecified outcomes, even if the intervention worked (or failed) in a potentially identifiable subset of trial participants. Post hoc analyses may predict nuances of trial design or trial participants who did or did not respond to the intervention, leading to testable hypotheses for refined enrollment and treatment strategies in subsequent prospectively designed streamlined trials. Such analysis or further perspectives will be of interest to the journal.

Precision medicine strategies (*e.g*., delivering the “right intervention to the right patient at the right time”) will likely be necessary for treating identifiable disease subtypes. We should expect that carefully designed enrollment of appropriate participants into clinical trials of correspondingly appropriate therapeutic agents and/or non‐pharmacologic interventions will be needed to deliver successful outcomes. A side benefit of this approach will be an accelerating knowledge of disease mechanisms that will lead to broader successes across the spectrum of ADRD as initial results are analyzed and expanded. Submissions corresponding to this theme will be encouraged.

Combination therapies informed through precision medicine may also be critical, and include any permutation of lifestyle modifications, non‐pharmacologic interventions, and either novel or repurposed drugs. *TRCI* will encourage submission of articles discussing such trial designs and study outcomes.

Updated reviews and perspectives on the landscape of clinical trials in ADRD will continue to be welcomed.

## 3 DIVERSITY, INCLUSION, AND HEALTH EQUITY

Racial, ethnic, sex, gender, socioeconomic, and other social identities should be considered contributory components to disease biology. It is recognized that “pure” descriptors do not exist in reality. Rather, admixtures are interwoven, coupled with differences in risk factors and comorbidities, some of which are biological and others environmental. It is important to study social exposures and social–biological phenotyping to understand how these biological and social determinants contribute to the complexities of AD&RD, including impacts on disease onset, progression, and interventional outcomes. While the challenges that these issues impose on clinical study enrollment cannot be overstated, they need to be embraced by the AD&RD field to move us beyond the all too commonly expressed limitations in published studies, that they were performed on (for example), “highly educated non‐Hispanic whites of European descent.” In addition, it is an obligation of the AD&RD research field to confront these issues directly to deliver outcomes to those who have been under‐represented in clinical trials. For *TRCI*, these considerations should be incorporated in submissions of observational and interventional clinical studies, either addressing them with data or discussing them in principle with a similar philosophy as noted above for basic science submissions, that they explain how their outcomes will lead to subsequent hypothesis‐driven clinical development studies. Perspective papers will be welcomed on how we can deconstruct and address these complexities based on scientific methodologies across the life course and full extent of lived experience in the context of translational research ranging from basic science to clinical interventions.

## 4 OPERATIONAL CONSIDERATIONS

The *TRCI* Editorial Board is being strategically constructed to include individuals with a broad range of expertise across the field of translational research and clinical interventions in AD&RD. This extensive content knowledge will ensure the guidance and balance of editorial policies, peer reviews, and author feedback across the following topic areas (listed in non‐prioritized order): (1) translational basic science studies; (2) preclinical and clinical pharmacologic interventions; (3) psychosocial and non‐pharmacological interventions; (4) diversity, inclusion, and health equity in clinical investigations and outcomes; (5) psychiatry and clinical psychology; (6) biomarkers; (7) genetics and functional genetics; and (8) bioinformatics, data analytics, and statistics. As a further vision for the journal, special topics will be planned by the Editorial Board in discussion and collaboration with ISTAART PIAs for solicitation of targeted submissions. “Special topics” compendia will be compiled and edited by Guest Editors who will be nominated by (and work with) members of the Editorial Board, with preference for researchers in the early career stages who will find value in this exercise for their personal career development. Perspective papers and reviews that fall within the topic interests of the journal will also be welcomed.

In summary, *TRCI* recognizes its opportunity for positive impact on the field of translational research in AD&RD, defining components that need to be focused and integrated from basic science and social constructs through clinical interventions and outcomes. It further recognizes its obligation to serve as a platform for expression of ideas across a range of perspectives. We will welcome input in joining and building this dialog, requiring only that they are science‐based and can be justified as falling within the scope of the journal. We express in advance our gratitude to those who will submit manuscripts to *TRCI*, and particularly the generous reviewers who provide critiques and support for the journal's deliverables. And of course we thank our extended “family”—our colleagues at Wiley, the Alzheimer's Association, and the A&D journals’ editorial boards. Together, we will strive toward contributing to the ultimate objective of translational research in AD&RD—to bring forward interventions that have positive impacts on the lives of patients and their care partners.

Barry Greenberg, Editor‐in‐Chief, on behalf of the A&D‐TRCI Editorial Board.
